# Vanadium dioxide thin films integrated with printed circuit board enables low-cost, reconfigurable millimeter-wave devices

**DOI:** 10.1038/s44172-025-00506-2

**Published:** 2025-10-07

**Authors:** Amir Afshani, Wenqiang Xiang, Tarek Djerafi, Mohamed Chaker

**Affiliations:** 1 Énergie Matériaux et Télécommunications, Institut National de la Recherche Scientifique (INRS), 1650 Boul. Lionel-Boulet, Varennes, QC Canada; 2 Énergie Matériaux et Télécommunications, Institut National de la Recherche Scientifique (INRS), 800, De La Gauchetière W., Montréal, QC Canada

**Keywords:** Electronic devices, Electrical and electronic engineering

## Abstract

Millimeter-wave switches are essential for reconfigurable and adaptive communication systems, yet current solutions often face trade-offs between performance, scalability, and cost. Here we present a scalable, high performance and cost-effective approach to develop reconfigurable millimeter-wave substrate integrated waveguide (SIW) devices by integrating vanadium dioxide (VO₂) thin films with printed circuit board (PCB) technologies. The integration technique involves depositing VO₂ films on thin, flexible polymer substrates, which are then transferred and affixed to PCB circuits. The VO₂ is thermally activated and selectively doped to reduce power consumption depending on applications. Using experimental prototypes, we demonstrate several reconfigurable devices operating in the millimeter-wave band, including series and parallel switches and a reconfigurable hybrid coupler that transforms into dual through-line SIWs. Electromagnetic simulations and measurements validate the approach, revealing low insertion loss, good isolation, and broadband operation. This method simplifies fabrication and supports large-area integration, offering a practical route to scalable, low-cost, reconfigurable millimeter-wave components.

## Abstract (web summary)

Amir Afshani and colleague propose and demonstrate an efficient method to develop mmWave switches by integrating VO₂ thin films with PCB technology. The approach simplifies fabrication, supports scalability, and achieves good performance.

## Introduction

The demand for high-frequency communication systems that have multifunction and reconfigurability has grown, which makes reconfigurable millimeter-wave (mmWave) technology essential. Configurability will enable 6 G and next-generation wireless technologies to achieve higher bandwidth and data rates^1–5^. In this context, mmWave switches^6,7^ as the backbone of reconfigurable and multifunction mmWave architectures drive the need for novel, low-loss, low-cost switching techniques.

Vanadium dioxide (VO_2_) stands out as a unique material that exhibits an insulator-to-metal transition (IMT) around 68 °C, making it highly attractive for tunable and reconfigurable mmWave devices^8^. Such devices offer significant advantages including versatile control methods through thermal, electrical, or optical triggers, which provides high switching speeds, low power consumption, and compact size, making them energy-efficient and suitable for miniaturized designs. Additionally, VO_2_ switches are cost-effective to manufacture, and they are compatible with existing semiconductor and waveguide technologies, making them ideal for advanced reconfigurable and mmWave communication systems^9^. VO_2_-based reconfigurable mmWave components have been widely used in recent works^10^ to outperform traditional semiconductor switches due to their lower parasitic effects. Moreover, VO_2_ has found progressive application in reconfigurable intelligent metasurfaces. It has been utilized in Ka-band linear-to-circular polarizer converters to switch polarization states^11^, and as mmWave electromagnetic spike detectors^12^ leveraging the sensitivity of the IMT transition threshold to incoming waves. Moreover, significant research efforts^8,13^ have focused on reducing the transition temperature of VO_2_ thin films via doping, aiming to lower the power consumption required to activate the switch. However, this approach often compromises the On/OFF ratio, which is why pure VO_2_ remains prevalent in many circuit applications.

While previous studies have demonstrated high-performance VO_2_-based switches for chip and thin-film technology, there is a significant need to integrate these high-performance VO_2_ materials with printed circuit board (PCB) technology. PCB is a low-cost, ubiquitous fabrication method for many circuits in mmWave and higher frequencies, capable of handling much higher power signals than chips, and providing greater design flexibility for developing front-end passive circuits. At these high frequencies, signal losses due to the skin effect in metallic structures become pronounced. In this context, substrate integrated waveguide (SIW) technology emerges as a superior solution due to its low loss, high shielding, and high-power handling capabilities, making it an excellent platform for developing mmWave front-end circuits such as wideband delay lines^14^, antennas and/or array antennas^15–17^, and etc. While VO₂-based switches have been previously implemented using microstrip technology, their integration with substrate-integrated waveguide (SIW) on PCB remains unexplored.

**Fig. 1:**
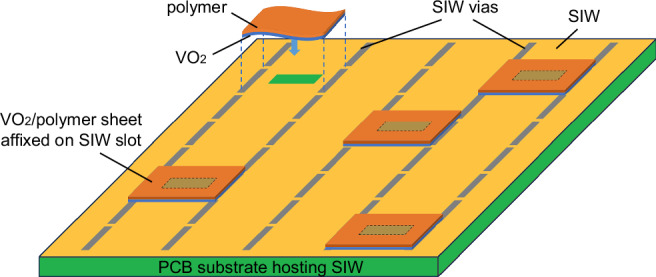
A conceptual illustration of the proposed method of combining VO_2_ thin films with SIW technology on PCB. Switching and reconfigurability can be achieved by affixing VO_2_ thin films to SIW circuits where required (SIWs are regions in PCB that is enclosed between vias). This technique enables low-cost, extremely low loss mm-Wave reconfigurable circuits with a simple fabrication process.

In this work, we present a novel approach that integrates thin-film VO₂ with PCB technology, leveraging the combined advantages of both technologies to develop low-loss, low-cost, and versatile mmWave switches with a simplified fabrication process. Unlike conventional semiconductor or MEMS-based switches, our methodology allows for seamless integration with standard PCB manufacturing, making it a highly scalable and practical solution for reconfigurable RF applications. Furthermore, we propose the first mmWave SIW switch based on VO₂ films, demonstrating its feasibility for high-frequency applications. By embedding VO₂ within the SIW structure, we achieve a compact, solid-state switching mechanism that benefits from SIW’s inherent shielding, reduced insertion loss, and improved power handling compared to microstrip-based implementations. Additionally, we explore the effect of tungsten doping in VO₂ as a means to modify its transition temperature, thereby reducing the thermal actuation power required for switching. Our approach is to deposit high-quality thin films of VO_2_ onto flexible and economical polymer substrates (Kapton) and then integrate these films into passive SIW circuits constructed with low-cost PCB technology, as shown in Fig. 1. The appeal of the proposed methodology lies in its potential for mass production, offering significant economic and industrial benefits. Prefabricated VO_2_/polymer sheets, prepared in advance in large batches or rolls with varying thicknesses and material properties, facilitate ease of use and extensive design options. Users can customize these sheets to the required dimensions and adhere them to designated areas on the PCB, enabling SIW switching functionalities through external thermal or electrical stimuli.

## Results and discussion

### Pure and tungsten doped VO2 thin films deposited on polymer substrates

**Fig. 2:**
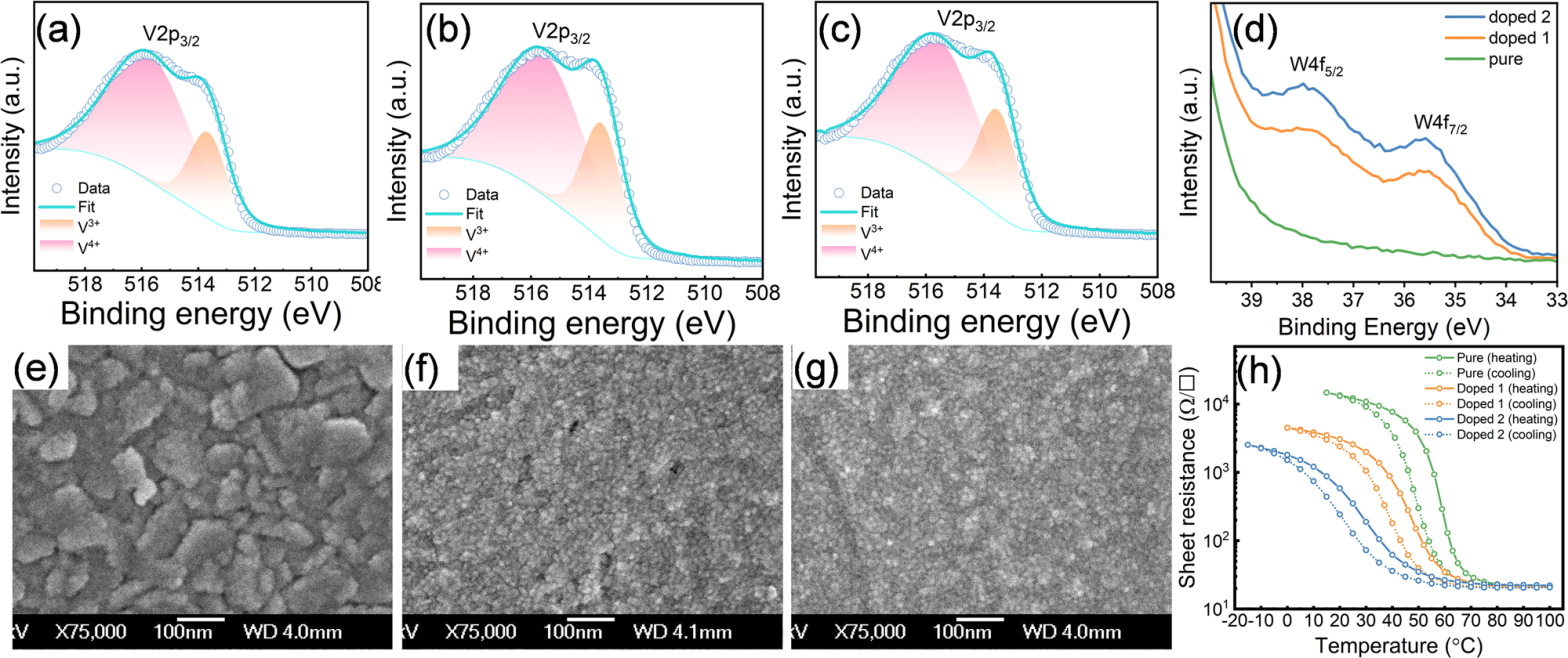
Characterization of VO_2_ thin films. XPS V2p₃/₂ spectra for **a** pure, **b** doped 1, and **c** doped 2 VO2 samples on Kapton. XPS W4f spectra for **d** pure, doped 1 and doped 2 samples. SEM top-view images of **e** pure, **f** doped 1, and **g** doped 2 samples. **h** Temperature dependent sheet resistance of pure, doped 1, and doped 2 samples.

The pure and W-doped VO_2_ films were synthesized on polymer substrates (Kapton HN) using pulsed laser deposition (PLD) as described in the methods section. The chemical compositions of pure and W-doped VO₂ samples were investigated using X-ray photoelectron spectroscopy (XPS), as shown in Fig. 2a–d. To minimize the effects of surface contamination and oxidation, all samples were etched with Ar ions for 6 min prior to XPS measurements. The deconvoluted V2p₃/₂ peak spectra for pure VO₂, 0.5 at% W-doped VO₂ (denoted as Doped 1), and 1.1 at% W-doped VO₂ (denoted as Doped 2) are shown in Fig. 2a–c, respectively. Among the XPS spectra, the peaks at 516.1 eV and 513.7 eV correspond to V⁴⁺ and V³⁺, respectively. The high proportion (> 70%) of V⁴⁺ in all three samples indicates the high quality of the VO₂ films. As the W-doping level increases from 0 to 1.1 at%, the V⁴⁺ ratio shows a slight decrease, from 76% for pure VO₂ to 73% for the Doped 2 sample. This reduction can be attributed to the substitution of V⁴⁺ by W⁶⁺ and changes in surface roughness due to element doping^18^. In Fig. 2d, the peaks at 37.9 eV and 35.6 eV correspond to W4f₅/₂ and W4f₇/₂, respectively, confirming the presence of W⁶⁺. By comparing the areas of the W4f and V2p peaks, the W doping concentrations for the Doped 1 and Doped 2 samples are calculated to be approximately 0.15 at% and 0.22 at%, respectively. However, given the resolution limit of XPS (~1%), we have used the W concentrations of 0.5 and 1.1 at% as determined by Rutherford backscattering spectrometry (RBS) in a previous work^19^. The surface morphology of the pure, Doped 1, and Doped 2 VO₂ films on Kapton is shown in Fig. 2e–g. As the W doping concentration increases from pure VO₂ to Doped 1 and Doped 2, the grain size of the VO₂ films decreases. This can be attributed to the changes in the microstructure induced by W incorporation. Specifically, the introduction of W creates defects that act as pinning centers, inhibiting further grain growth^20^.

The temperature-dependent sheet resistance for pure VO_2_, doped 1, and doped 2 thin films on polymer substrates is shown in Fig. 2h. For the pure VO_2_ sample, the sheet resistance ranges from 23 Ω/sq at 100 °C to 18135 Ω/sq at 20 °C, representing approximately 2.85 orders of magnitude in electrical contrast, with a transition temperature at 53 °C. For the doped samples, the sheet resistance in the metallic state remains similar to the pure VO_2_, around 23 Ω/sq. However, due to the additional charge carriers introduced by W doping^21^, the sheet resistance in the semiconductor state is lower compared to the pure sample. Moreover, due to the combined effects of element doping and substrate-induced strain^19^, the transition temperatures for the doped samples are lowered to 42 °C for doped 1 and 24 °C for doped 2. A more detailed discussion on the sample’s transition temperatures can be found in previous paper^19^.

### Millimeter-Wave SPSD switch

#### In-series configuration

**Fig. 3:**
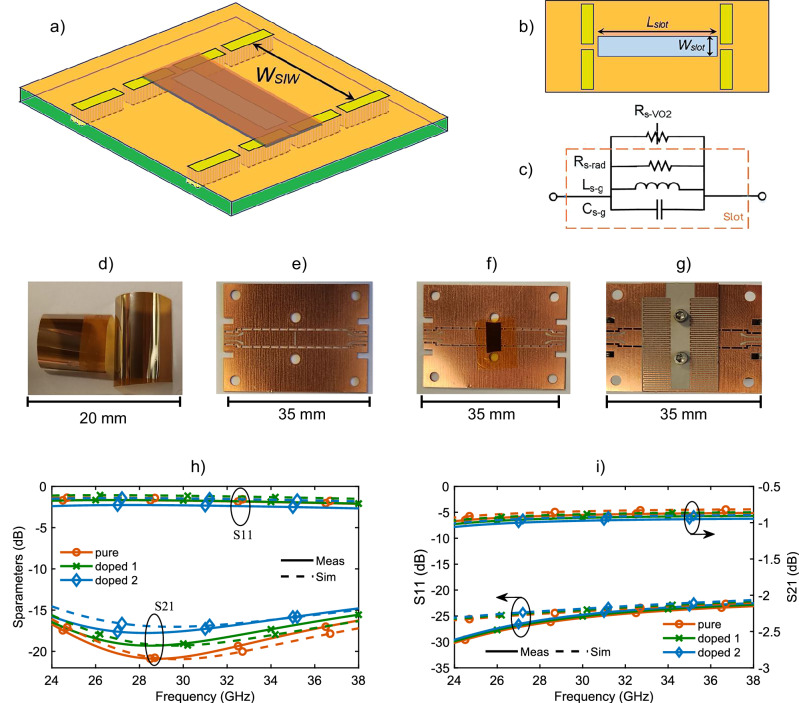
MmWave SIW switch based on VO_2_ in series configuration. **a** Perspective view of schematic of the proposed switch in series configuration. **b** Top view showing dimensions of the slot: *WSIW* = 4.5 mm, *Lslot* = 4.1 mm, *Wslot* = 0.8 mm. **c** Lumped circuit model of the switch. **d** VO_2_/polymer thin films prepared using PLD technique. **e** fabricated SIW on PCB. **f** VO_2_/polymer taped on SIW to function switching. **g** meander line heater fabricated on another PCB, which sits on top of the SIW/VO_2_/polymer layers to activate the switch. **i**–**h** S-parameter measurement and simulation results for mmWave SIW switch based on VO_2_ in series configuration at: **i** Low temperature, **h** High temperature.

The schematic of the proposed mmWave switch in series configuration is shown in Fig. 3a. The switch comprises a PCB layer at the bottom and a VO_2_/polymer layer on the top of PCB. The PCB embodies the substrate integrated waveguide (SIW) with a transverse slot etched on its top copper wall. SIW is formed by creating two rows of metalized vias on the PCB, which effectively act as waveguide wall^22^. VO_2_/polymer lays on the SIW covering the slot, with VO_2_ layer facing the SIW and in direct contact with copper of the PCB surrounding the slot. The fabrication process is discussed in the methods section of this paper.

A Rogers 6002 substrate with a dielectric constant of 2.94, a loss tangent of 0.0012, and thickness of 0.5 mm forms the PCB layer. Although the proposed design methodology is scalable to different frequencies and PCB materials, we have selected this substrate due to its excellent temperature stability.

The cut-off frequency of the SIW is determined from^22^

$${f}_{c}=\frac{c}{2{W}_{{SIW}}\sqrt{\varepsilon r}}$$

where *c* is the speed of light at the free space, *W*_*SIW*_ (*According to* Fig. 3) is the SIW width and *ε*_*r*_ is the dielectric constant of the substrate. For mmWave operation, the width of SIW is chosen as 4.5 mm which results in a cut-off frequency of 19.45 GHz. Considering the second mode cut-off frequency at 38.9 GHz and some safe frequency guard, the switch operation range is set from 24 GHz to 38 GHz.

Slot etched on the SIW creates discontinuity for the current flowing on top wall. Therefore, a relatively large slot on top of the SIW results in substantial wave reflection at the input port due to impedance mismatch. At low temperature, VO_2_ acts as a dielectric and has a partial loading effect on the slotted SIW. However, at high temperatures, VO_2_ transitions into a metallic state, allowing it to conduct current on the top wall and effectively reduce the current discontinuity. As a result, the wave propagates effectively through the SIW at high temperatures, with only minimal insertion loss from the limited conductivity of VO2.

Figure 3c illustrates and models the switch with circuit lumped element at low and high temperatures. The slot is modelled with a capacitor and inductor due to the parasitic effects of the slot on the current. Also, the slot exhibits radiation and ohmic losses due to the VO_2_conductivity, which is considered as made of two resistors. At low temperature, ohmic losses are negligible and the parasitic effects dominate the switch performance. The larger the slot, the larger the parasitic effects. The inductance of the slot yields a larger impedance mismatch and wave reflection at the input. In contrast, at high temperature, the parasitic effects are mitigated and the ohmic loss of VO_2_ dominates. Therefore, the high-temperature conductivity of VO_2_ determines the insertion loss of the switch.

We should mention that, to design the switch, we have exploited CST Microwave Studio to simulate the electromagnetic interactions within the circuits and to optimize the dimensions of the circuits. More information regarding the simulations is presented in the methods section of this paper. All measurements presented in this paper are in agreement with the simulation results, which support the theoretical foundation of this work.

The mmWave VO_2_/SIW switch is fabricated and prepared as shown in Fig. 3d–g. For better contact with the VO_2_ with the SIW, VO_2_ is affixed with a Kapton tape to the SIW. To heat VO_2_, we have considered two approaches. In one of them, heating is achieved externally. In the second approach, a heater with copper meander lines is designed on another thin PCB sitting on the VO_2_/polymer. The heater converts the electric current into heat using Joule effect to induce the insulator to metal transition of VO_2_. Both approaches yield similar results at low and high temperature, offering two effective techniques to induce the switch. Although VO₂ materials have the potential for ultra-fast switching when activated optically or via laser, the switching speed in our study is relatively slow due to thermal activation. The measured activation time is approximately 10 se. Achieving ultra-fast switching for large-area VO₂ samples remains an open challenge and could be explored in future studies.

The measurement results are shown in Fig. 3h–i. For measurement purpose, we have adopted Co-Planar Waveguide (CPW) at device terminals. To match SIW to CPW ports taper transitions are used which gradually transform large impedance of the SIW to the 50-ohm impedance of CPW at the operation frequency band. For accurate measurements TRL calibration has been used to eliminate the effect of the SIW to CPW transitions as well as the connection cables and connectors. The TRL calibration kit is briefly presented in the methods section of this paper.

Measurements are conducted for three different VO_2_ samples: Pristine VO_2_/polymer (pure VO_2_), 0.5 at% W-doped VO_2_ (doped 1), and 1.1 at% W-doped VO_2_ (doped 2). Figure 3h, i show the S-parameters of the switch at low and high temperature, respectively, for three samples. Measurement results (in solid lines) are in excellent agreement with the simulation results (in dashed lines) validating the proposed device and the fabrication technique.

Figure 3h shows that the SIW switch effectively blocks wave propagation at low temperature as the reflection coefficient (S_11_) is weak and the transmission coefficient/isolation (S_21_) is large. (the coefficients in the figure are in logarithmic scale). The S_21_ at low temperature indicates the isolation level of the switch, which is better than 15 dB almost over the whole band for all three samples. However, it is noted that by increasing the percentage of tungsten dopant in the VO_2_ sample, the isolation slightly drops, which is expected as the low-temperature conductivity of VO_2_ is being increased. On the other hand, at high temperature, Fig. 3i indicates a low-loss performance of the switch for all samples. The insertion loss (S_21_) is less than 1.1 dB for all samples, which is excellent, and the switch is well matched over the whole operation band. Since at high temperature all the VO_2_ samples exhibit a similar resistivity (Fig. 2h), the switch performance at high temperature is similar for all three samples. In the proposed design, the insertion loss and isolation in the in-series configuration are highly dependent on the slot width, exhibiting a see-saw relationship. To achieve better isolation, the slot width should be increased, although this will result in a slight increase of insertion loss.

Therefore, from S-parameter measurements, it can be concluded that all three VO_2_ samples can be used as active element for efficient series mmWave switch, even though higher W-dopant concentrations lower the switch isolation at low temperature. However, as far as power consumption is concerned, the switch with higher W-dopant concentration has a lower transition temperature and hence requires lower activation energy. Therefore, the trade-off between isolation in the OFF state and the power consumption in the ON state determines which VO_2_ sample is the best in specific applications.

**Fig. 4:**
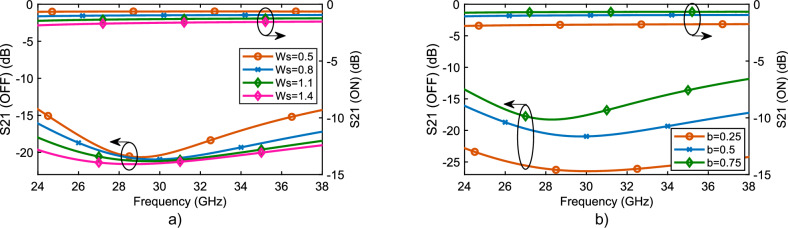
Simulated parametric analysis of the series configuration. **a** Comparison of S_21_ in the OFF and ON states for four different slot widths. As observed, increasing the slot width results in higher isolation and insertion loss. **b** Comparison of S_21_ in the OFF and ON states for three different PCB substrate thicknesses. The results indicate that decreasing the PCB thickness increases both isolation and insertion loss.

To evaluate the performance of the proposed switches, we conducted a parametric sweep on key dimensions of the SIW switch in the series configuration. The two primary parameters analyzed were the slot width (Ws) and the SIW/PCB thickness (b). The simulation results are presented in Fig. 4. Figure 4a shows that increasing the slot width leads to a simultaneous increase in both insertion loss and isolation, which aligns with expectations. In this design, we selected a slot width of 0.8 mm to achieve at least 16 dB isolation at the band edges and up to 21 dB at the center of the band. Figure 4b illustrates the impact of varying the SIW thickness. As shown, reducing the PCB thickness (b) increases both insertion loss and isolation. To balance these trade-offs, we chose a thickness of 0.5 mm as an optimal compromise.

Overall, the results in Fig. 4 demonstrate the design flexibility of the proposed switch, allowing for higher isolation levels if required—albeit at the expense of increased insertion loss.

#### In-parallel configuration

**Fig. 5:**
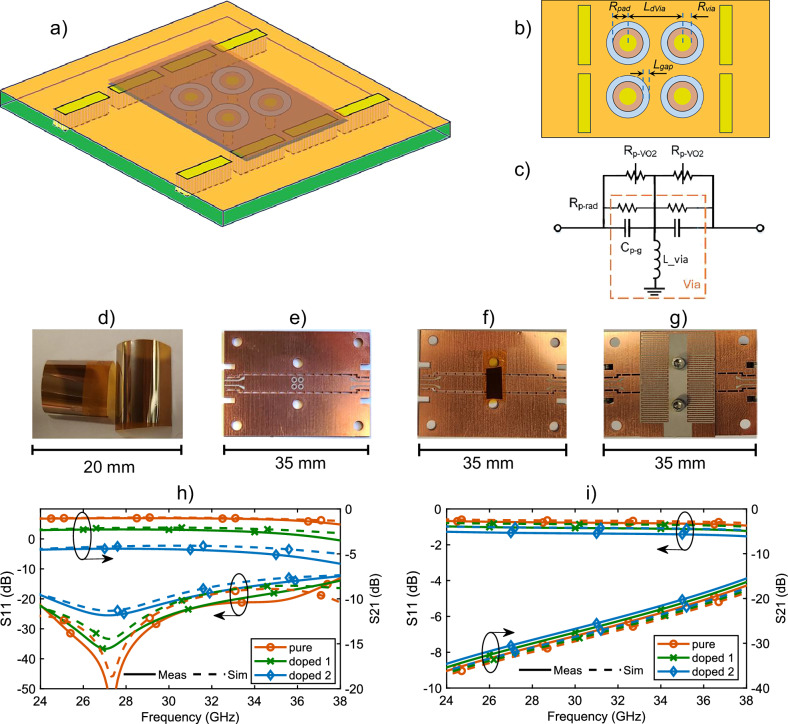
MmWave SIW switch based on VO_2_ in parallel configuration. **a** Schematic of the proposed switch in parallel configuration. **b** Top view showing dimensions of: *Lgap* = 0.3 mm, *Rvia* = 0.2 mm, *Rpad* = 0.5 mm, *LdVia* = 1.9 mm. **c** Lumped circuit model of the switch. **d** VO_2_/polymer thin films prepared using PLD technique. **e** fabricated SIW on PCB. **f** VO_2_/polymer taped on SIW to function switching. **g** meander line heater fabricated on another PCB, which sits on top of the SIW/VO_2_/polymer layers to activate the switch. **h**–**i** S-parameter measurement and simulation for results mmWave SIW switch based on VO_2_ in parallel configuration at: **h** Low temperature, **i** High temperature.

The schematic of the proposed mmWave VO_2_/SIW switch in parallel configuration is shown in Fig. 5. The layers configuration is the same as the in-series configuration. The difference is that four vias with VO_2_-covered pads are replaced with the slot at the center of the SIW. At high temperature, VO_2_ is in its metallic state and short circuits the pads of the vias, connecting the top and bottom conductors of the SIW. Since the current flows between top and bottom conductors though the four vias, the admittance of the SIW drastically changes and the wave is reflected at the input port due to significant impedance mismatch. However, at low temperature, VO_2_ is in its dielectric state, and therefore, the current cannot flow from top to bottom conductors. On the other hand, since the size of vias and pads are chosen relatively small, they induce negligible perturbation to the current flow, allowing the wave to propagate though SIW with low insertion loss.

The parallel configuration is modeled in Fig. 5c. Each via can be modeled with an inductor and capacitor for current perturbation and capacitive effect, respectively, between two conductors. Additionally, the pads exhibit negligible radiation loss and contribute ohmic loss from the VO_2_, both of which are modeled separately using lumped resistors. In this configuration, pads’ conductivity plays a decisive role in the performance of the switch. At low temperature, since the size of the pads is small, their conductivity, even low, can contribute to a noticeable current flow from top to bottom conductor through the vias and degrade the insertion loss. At high temperature, the isolation of the switch depends on its conductivity; hence, a large conductivity is required.

The switch in parallel configuration was fabricated and prepared as shown in Fig. 5d–g. The VO_2_/polymer is taped to SIW using a Kapton tape for better contact between two layers. The measurement results are displayed in Fig. 5h, i for three samples, where they are in great agreement with the simulation results. At low temperature (Fig. 5h), the switch is well matched for all three samples. However, as discussed and expected before, pure VO_2_ exhibits excellent insertion loss of about 1.0 dB. As the percentage of tungsten dopant increases and the low-temperature conductivity of the VO_2_ sample increases, the insertion loss of the switch deteriorates. At the center frequency, it is 2.3 and 4.5 dB for doped 2 and doped 3 samples, respectively. At high temperature (Fig. 5i), the switch stops wave propagation with a relatively large isolation better than 16 dB at high frequency and better than 34 dB at low frequency in the operation band. Since the resistivity of VO_2_ at high temperature is similar for all three samples, their S-parameter response is also similar in the ON state of the switch.

While a similar parametric analysis could be performed for the parallel configuration, it is omitted here to maintain clarity and focus. Unlike the series configuration, the performance of the parallel switch does not scale directly with substrate thickness. Instead, the via and pad dimensions must be re-optimized for each substrate thickness. Our simulations confirm that comparable performance can be achieved for substrate thicknesses of 0.25 mm and 0.75 mm when properly optimized.

### Thermal analysis

**Fig. 6:**
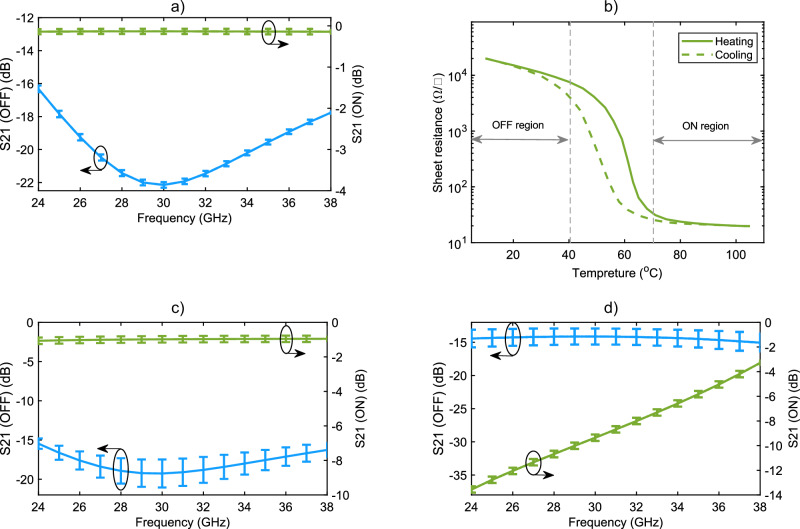
Measured temperature sensitivity analysis of the switches. Error bars indicate mean ± standard deviation computed across 40 temperature points per region (10–40 °C OFF and 70–110 °C ON), combining 20 heating and 20 cooling measurements at each frequency. **a** Temperature analysis of the PCB without VO₂. The PCB with a slotted SIW was measured at various temperatures from 10 °C to 110 °C, with the isolation plot shown in blue along with error bars. Similarly, a PCB with a SIW thru line was measured at the same temperature points, with the insertion loss plot shown in green. Measurements indicate that the PCB itself is highly stable and resistant to temperature variations. **b** Resistivity curve for pure VO₂, extracted from Fig. 2h, used to analyze the temperature-dependent performance of the switches. Based on this, two temperature regions were defined: the OFF region (10–40 °C) and the ON region (70–110 °C). The switches were measured at 20 temperature points in each heating and cooling cycles. **c** Measured isolation and insertion loss for the series configuration at OFF and ON states, with error bars. The results indicate that temperature sensitivity primarily affects isolation, though it remains better than 15 dB across the band. **d** Measured isolation and insertion loss for the parallel configuration at OFF and ON states, with error bars. The results show that temperature sensitivity has a more pronounced effect on insertion loss in this configuration.

In this section, we investigate and measure the temperature sensitivity of the proposed switches. To do so, we first assess the thermal behavior of the PCB and then focus on the VO₂ material. Figure 6a examines the temperature sensitivity of the PCB by measuring the SIW thru-line and slotted SIW lines (series switch without VO₂) at 20 temperature points within the 10–110 °C range. The green curve and error bars represent the average insertion loss and its variation range for the SIW thru-line, respectively. Similarly, the blue curve represents the average isolation and its variation range for the slotted SIW line. Both curves indicate that S_21_ variations due to temperature changes are minimal and negligible.

Figure 6b–d focus on the temperature sensitivity of VO₂. Figure 6b replots the resistivity vs. temperature hysteresis curve for the pure VO₂ sample from Fig. 2h. As seen, VO₂ resistivity exhibits significant variation within the 40–70 °C range during both heating and cooling cycles. For stable operation, the VO₂ switch must be used outside this transition region. Although extreme temperatures (such as 20 °C and 100 °C) provide the best performance, a broader operational temperature range can be defined where the switch still functions effectively. Based on this, temperature ranges of 10–40 °C and 70–110 °C are designated as the OFF and ON regions, respectively.

To evaluate the impact of temperature variations, we measured the series and parallel switches at 40 temperature points within these regions during both heating and cooling cycles. The results are presented in Fig. 6c, d for the series and parallel configurations, respectively. These figures show that temperature sensitivity is more pronounced in the OFF state for both configurations (blue curves). However, even in the OFF state, the series switch maintains an isolation of more than 15 dB, and the parallel switch maintains an insertion loss of less than 2.1 dB across the entire band. To further reduce temperature sensitivity, the OFF region can be shifted to a lower temperature range. In the ON state, temperature variations in both configurations (green curves) remain minimal, demonstrating good thermal stability of VO₂ above 70 °C.

### Millimeter-Wave reconfigurable SIW hybrid coupler/Dual SIW through line

The proposed technique for realizing a SIW switch based on VO_2_ can also be applied for developing other reconfigurable mmWave components as well. As an example, here we investigate the realization of a reconfigurable mmWave hybrid coupler at room temperature which transforms into a dual parallel SIW through line at high temperature. A hybrid coupler is a reciprocal four-port component that splits the input signal at port 1 equally between ports 2 and 4 with a 90-degree phase difference, while port 3 remains isolated from port 1. This distribution pattern holds for signals input at the other ports as well, ensuring high isolation and consistent signal distribution.

**Fig. 7:**
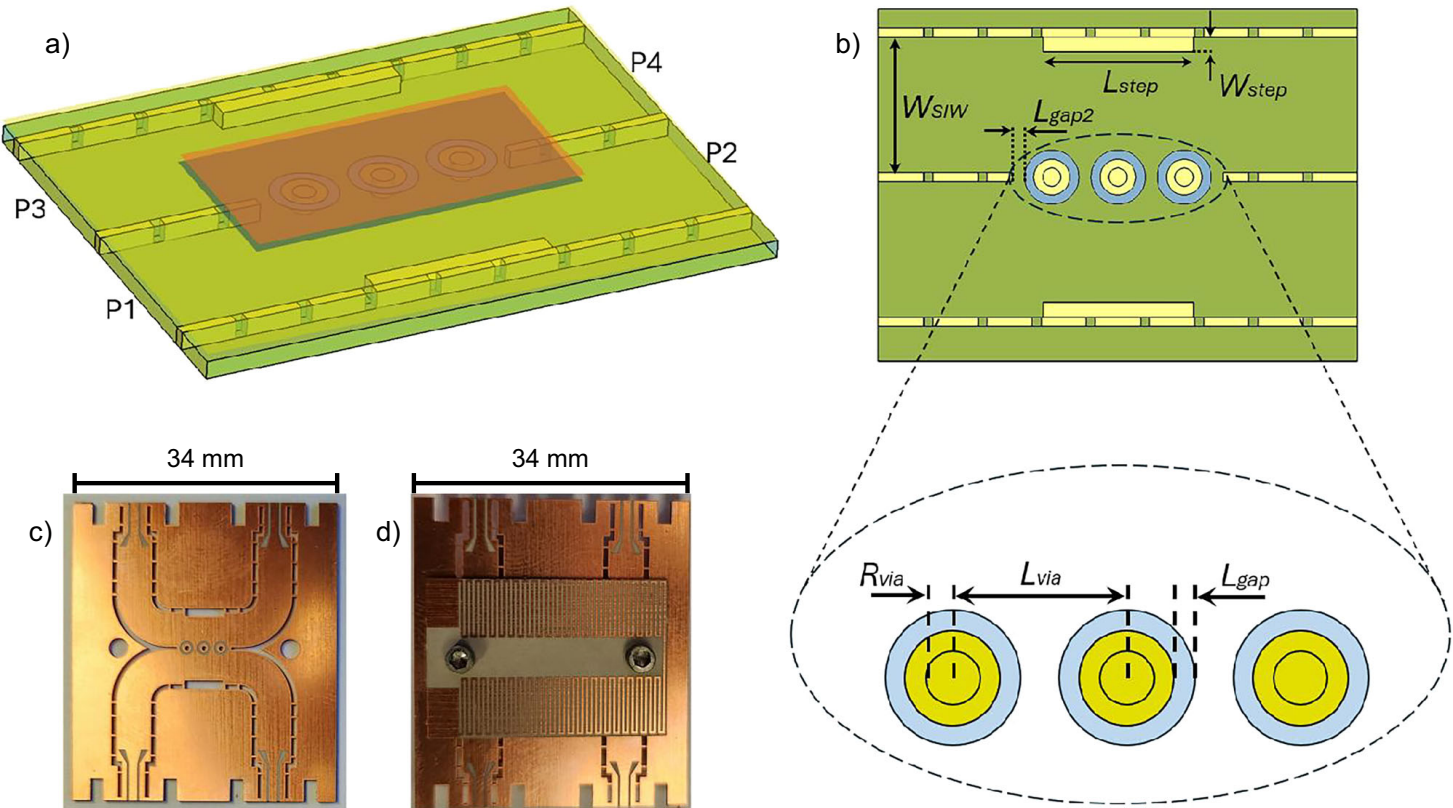
MmWave reconfigurable SIW hybrid coupler/ Dual SIW through line, based on VO_2_. **a** Perspective, **b** top view of the proposed reconfigurable component with dimensions of: *Lgap* = 0.3 mm, *Lgap2* = 0.4 mm, *Rvia* = 0.2 mm, *Lvia* = 2.2 mm, *Lstep* = 5.0 mm, *Wstep* = 0.5 mm, and *Wsiw* = 4.5 mm. **c** fabricated prototype with SIW to CPW transitions. **d** fabricated prototype with PCB integrated heater sitting on top of the circuit.

The schematic of the proposed component is illustrated in Fig. 7a, b. To design the proposed reconfigurable component, a SIW hybrid coupler was first designed at 28 GHz. It consists of two parallel SIWs which are coupled through a gap in the common wall between them. The size of this gap is chosen according to the desired coupling between the two SIWs (3 dB in this case). In the coupling region, a narrow-width step is introduced for two reasons: first, to serve as an H-plane impedance step for impedance matching, and second, to suppress the unwanted TE_30_ mode by reducing the waveguide width. For reconfigurability, three vias with VO_2_-covered pads (similar to vias used in the parallel switch) are placed in the coupling gap along the common wall of the SIWs. These vias have a negligible effect at low temperature and do not perturb the hybrid coupler, while at high temperature they connect top and bottom conductors of SIW and therefore stop wave propagation. As a consequence, at high temperature, these vias turn into an effective wall filling the coupling gap between SIWs and resulting in two uncoupled parallel through line SIWs. The image of the fabricated reconfigurable mmWave component is displayed in Fig. 7c. In the fabricated prototype, since the device size is small, 90-degree SIW bends are used to put SIW terminals far from each other, which has a negligible effect on the performance of the proposed component. For measurement purpose, SIW to CPW taper transitions are used to effectively match SIW to 50-ohm CPW lines over the whole operation band. Furthermore, to apply voltage stimulus to VO_2_ films, a heater is designed on another thin PCB which stands on the VO_2_/polymer film, as shown in Fig. 7d.

**Fig. 8:**
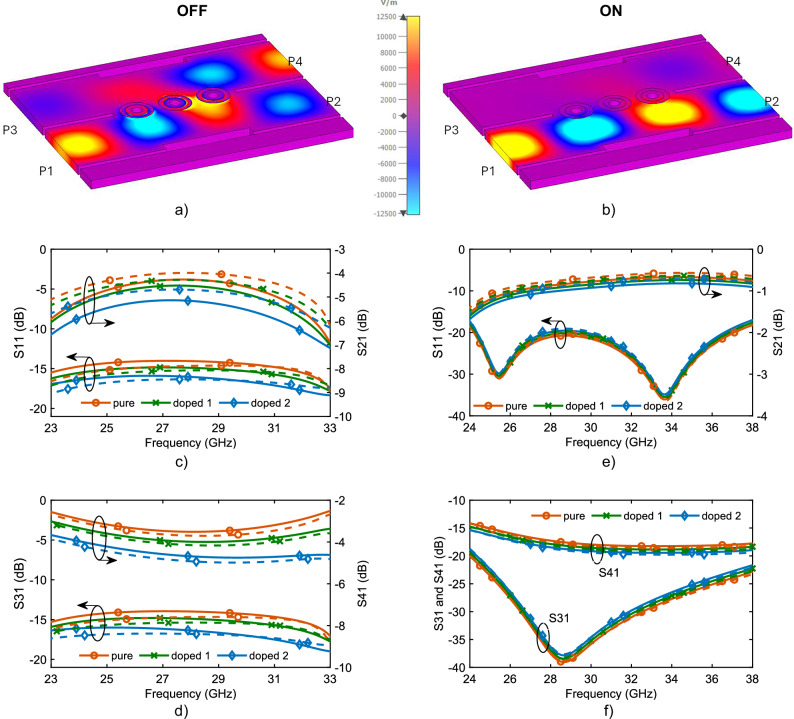
Measurement and simulation results for mmWave reconfigurable SIW hybrid coupler/ Dual SIW through line. Simulated electric (E) field patterns of the proposed component when port 1 is exited at (**a**) low, and (**b**) high temperature. S-parameter results at the (**c**), (**d**) low, and (**e**), (**f**) high temperatures. These results demonstrate a successful transition from SIW hbrid coupler to dual SIW through lines, when switch is activated.

To better illustrate the operation of the proposed reconfigurable component, Fig. 8a, b show the electric field patterns at low and high temperatures, respectively, when port 1 is excited. This figure shows that at low temperature, wave input at port 1 is transmitted into port 2 and 4 with almost equal amplitude but 90-degree phase difference, while port 3 is uncoupled and isolated (low leakage observed from port 1 to port 3). However, at high temperature, as shown in Fig. 8b, the wave input at port 1 is transmitted to port 2, with slight leakage to port 3 and 4, indicating that two SIWs are highly uncoupled and well isolated.

The measurement results are shown in Fig. 8c–f in solid lines and are compared to the simulation results in dashed lines for the three VO_2_ samples with different tungsten doping concentrations. Figure 8c, d present the simulation and measurement results at low temperature, validating the operation of the hybrid coupler when port 1 is excited. Figure 8c shows that the device exhibits a reflection better than 15 dB, indicating good matching over the whole operation band. Furthermore, Fig. 8c reports forward coupling from port 1 to port 2 on the right axis. Simulation and measurement results follow the same trend, even though measurements yield about 0.5db more insertion loss, which can be attributed to the fabrication tolerance and imperfect contact of VO_2_ to SIW. For the pure VO_2_ sample, the measured forward coupling is about −4.2 dB which shows about −1.2 dB insertion loss considering a perfect 3 dB splitting power. However, the insertion loss increases for the other samples and as tungsten doping concentration increases, as expected and in line with the observations in previous section for parallel switch. In the worst sample of 1.1 atomic % W-doped (doped 2), the insertion loss reaches −5.1 dB at the center frequency of 28 GHz. Figure 8d reports the results for the isolated and coupled ports of 3 and 4, respectively, when port one is excited. In this figure, measurements and simulations again follow a similar trend, even though measurements indicate about 0.5 dB less insertion loss than simulations, which is consistent with the increased insertion loss at the forward coupling in Fig. 8c. For pure VO_2_, insertion is about −3.5 dB, while it increases to −4.0 dB and −4.6 dB for doped 1 and 2 samples, respectively. Since the device is optimized to accommodate all three VO₂ samples simultaneously, a slightly elevated amplitude imbalance is observed. Nevertheless, it remains within an acceptable range of ±0.8 dB across 25–31 GHz for all cases and can be further minimized by tailoring the design to each VO₂ sample individually. Furthermore, this figure shows that the device isolation is better than 14 dB (S_31_) for all samples over the whole frequency band.

Figures 8e, f report the results at high temperature, when the component transforms into dual SIW through lines. Simulations and measurements are in excellent agreement for all three samples. Figure 8e shows the reflection coefficient on the left axis, which indicates a great impedance matching from 24 to 38 GHz. Furthermore, this figure presents an excellent insertion loss from port 1 to port 2, which is better than 1 dB for most of the upper band and drops at lower frequency as it approaches the cut-off frequency, as expected. Since at high temperature the resistivity of three samples is similar, the corresponding results for three samples are similar as well. Figure 8f reports the coupling from port 1 to ports 3 and 4, which are below 15 dB (almost 17 dB from 28 to 38 GHz) and 20 dB, respectively, over the whole band from 24 to 38 GHz. The proposed reconfigurable device exhibits lower insertion loss at higher frequencies compared to lower frequencies for two main reasons. First, in the ON state, the component demonstrates slightly better isolation at higher frequencies, resulting in reduced power leakage to the isolation port. Second, the designed SIW has a cutoff frequency of approximately 19.45 GHz; as the operating frequency approaches this threshold, wave propagation becomes less efficient, leading to increased attenuation and higher insertion loss. This figure shows that two SIWs are well isolated from each other and constitute a dual SIW though line with low insertion loss, specifically for the pure VO_2_ sample. Overall, results in Fig. 8 demonstrate the successful achievement of a reconfigurable mmWave component which transforms from a hybrid coupler into a dual SIW through line with excellent performance and low insertion loss.

The proposed series and parallel SIW VO₂ switches are compared to state-of-the-art mmWave waveguide switches based on different technologies in Table 1. PIN diode-based waveguide switches^23,24^ offer fast switching speeds, making them a convenient option for mmWave applications. However, their RF performance is often compromised, either by low isolation^23^ or excessively high insertion loss^24^. Photoconductive SIW switches^25–27^ utilize photoconductive elements whose conductivity is modulated by light, enabling very fast optical activation. Despite this advantage, they suffer from a complex fabrication process and exhibit either limited bandwidth^25,26^ or high insertion loss^26,27^. Mechanical SIW switche^28^ provides exceptionally high isolation (>45 dB) but suffers from a high insertion loss of approximately 4.5 dB. Its key advantage is the ability to handle high-power signals better than most other technologies, though its slow and inconvenient switching mechanism remains a significant drawback. MEMS-based waveguide switches^29,30^ offer excellent RF performance, with low insertion loss and relatively good isolation. However, they require a complex and costly fabrication process, making them less practical for cost-sensitive applications. Liquid metal^31^ and microfluidic-based^32^ switches present another alternative for mmWave applications. These technologies provide low insertion loss with moderate isolation, but liquid metal switches require a cumbersome pumping mechanism and suffer from slow switching speeds. While there is limited paper on mmWave SIW switches based on liquid metals, most existing works focus on lower frequency bands^33^.

**Table 1:** Performance comparison of mmWave waveguide switches

work	RF Performance				Technology/ Activation method	Pros	Cons
	Freq. (GHz)	BW (%)	IL. (dB)	Iso. (dB)			
[23]	20 - 25	22	2.0	10	Pin diode/ Voltage	- Simple and low-cost fabrication - Fast switching	- High insertion loss - Low isolation - Low bandwidth
[24]	69 - 90	26	4.0	25	Pin diode/ Voltage	- simple and low cost - Fast switching	- Very high insertion loss - Low bandwidth
[25]	23-26.5	14	1.0	17	Photoconductive/ Optical	- Fast switching	- Low bandwidth - Complex fabrication
[26]	72-89	21	2.0	25	Photoconductive/ Optical	- Fast switching - W band	- High insertion loss - Low bandwidth - Complex fabrication
[27]	28–47	53	2.5^a^	25	Photoconductive/ Optical	- Fast switching - Large bandwidth	- High insertion loss - difficult fabrication - Complex fabrication
[28]	50–75	40	4.5	45	Mechanical	- Very High isolation - High power handling - Large bandwidth	- Very High insertion loss - Mechanical switching - Very slow switching
[29]	60-75	22	0.2	22	MEMS/ Voltage	- Very low insertion loss	- Low bandwidth - Complex fabrication
[30]	60-70	15	0.4	30	MEMS/ Voltage	- High isolation	- Very Low bandwidth - Complex fabrication
[31]	18-26	36	0.5	15	Liquid Metal/ Pumping	- Low insertion loss	- Low isolation - Inconvenient biasing - Very slow switching
[32]	25-40	46	0.5	22	Microfluid/ Pumping	- Low insertion loss - Large bandwidth	- Low isolation - Inconvenient biasing - Very slow switching
This work (SPST series, Pure VO_2_)	24-38	45	0.8-0.9	16-21	VO_2_ on PCB/ Thermal-Voltage	- Simple and low-cost fabrication - High power handling - Large bandwidth	- Slow switching
This work (SPST parallel, Pure VO_2_)	24-38	45	0.8-1.2	19-36	VO_2_ on PCB/ Thermal-Voltage	- Simple and low-cost fabrication - High power handling - Large bandwidth	- Slow switching

*Freq Frequency, *BW* bandwidth, *IL* Insertion Loss, *Iso* Isolatio, *Tech* Technology.

^a^Maximum IL is an estimation from figures as only peak IL is reported. Also, TRL calibrations are not used to de-embed the transition effect.

In comparison to the existing waveguide switch technologies summarized in Table 1, the proposed SIW VO₂ switches offer an exceptionally wide bandwidth while maintaining relatively low insertion loss and good isolation, outperforming several other solutions. Furthermore, the VO₂-polymer on PCB technology enables a cost-effective and straightforward fabrication process. Given these advantages, our approach presents a highly promising solution for the development of mmWave SIW switches.

## Conclusion

In this paper, we proposed a novel approach to combine PCB and thin film fabrication technologies to develop a low-cost mmWave switch with easy fabrication process and excellent quality. The proposed approach involves developing thin film VO_2_ on low cost thin and flexible polymer substrates such as Kapton. The prepared VO_2_/polymer sample can be then integrated to PCB where required as a reconfiguring or switching element. To validate the proposed approach, we have developed and demonstrated three mmWave components with excellent measurement results that are in great agreement with simulation results. The first and second components are mmWave SIW switches in series and parallel configurations, respectively. While the first one allows the wideband mmWave wave flow at high temperature, the second configuration allows mmWave wave flow at low temperatures.

The third demonstration is a reconfigurable passive mmWave component which acts as a SIW hybrid coupler at low temperature, while transforming into a dual SIW through line at high temperature.

All three components demonstrated excellent wideband performance with lower insertion loss. The low loss, compact size, and planar form of this reconfigurable approach make it easy to integrate into microwave and millimeter-wave planar circuits.

Furthermore, we have explored the effect of tungsten doping on VO_2_ thin films to tune the transition temperatures. Tungsten doping lowers the transition temperature, which reduces power consumption for switching, though it comes with a slight increase in loss. Therefore, depending on the application, one may choose the appropriate doping concentration required to either optimize performance or power consumption. More importantly, since this trade-off is determined solely by the VO_2_/polymer layer, different VO_2_-W doped/polymer substrates can be used with the same PCB circuit. This significantly reduces and simplifies both the fabrication cost and process.

## Methods

### PCB fabrication

The module has been fabricated using standard printed circuit board (PCB) technology. The PCB substrate is Rogers RT/duroid 6002 laminate, with a dielectric constant of 2.94 and a loss tangent of 0.0012. All vias were created within the substrates with the patterned laser ablation of the substrate and metalized with electroplating technique.

### VO_2_ thin films deposition

The pure and W-doped VO_2_ films on polymer substrates (Kapton HN) were synthesized using a combination of pulsed laser deposition (PLD) and an oxidation coupling technique. Initially, VO_*x*_ and W-doped VO_*x*_ films were deposited onto the polymer substrate by PLD. Two Kurt J. Lesker targets were utilized: pure vanadium and 1.5 at. % W-doped vanadium. In addition, V foil was added on top of the W-doped target to achieve different doping concentration. Depositions were carried out at room temperature, using a 248 nm KrF excimer laser with a repetition rate of 10 Hz. The laser fluence on the targets was maintained at 2 mJ·cm⁻², and the oxygen pressure was kept at 5 mTorr. The thicknesses of the VO_*x*_ films were 40 nm, based on test depositions performed on a Si substrate to determine the deposition rate. Post-deposition, the samples were annealed at 390 °C for 2.9 hours, with an oxygen pressure of 70 mTorr, resulting in a final film thickness of 50 nm.

### Simulation

The module’s design and full-wave electromagnetic simulations were done in the commercial software CST Microwave Studio using frequency-domain solver that is based on finite element method (FEM). Since VO_2_ displays two different behaviors at low and high temperatures, we considered two separate models to simulate vanadium dioxide (VO_2_) at low and high temperatures. At high temperature, since VO_2_ is in metallic state and has very high conductivity, we used an ohmic sheet to model the VO_2_. The resistance of the ohmic sheet was set as the sheet resistance of the prepared VO_2_ thin films which were measured using a four-point probe. At low temperature, the VO_2_ was modelled with a lossy dielectric layer. The conductivity of the VO_2_ layer was set based on the measured conductivity of prepared VO_2_ samples using the four-point probe technique.

### Measurement

Rutherford backscattering spectrometry (RBS) was used to determine the W concentration in the films. The surface morphology of the thin-film samples was examined using scanning electron microscopy (SEM). Temperature-dependent sheet resistance measurements were performed using a 4-point probe station equipped with a heating/cooling module. The chemical composition of the thin films was analyzed via X-ray photoelectron spectroscopy (XPS).

**Fig. 9:**
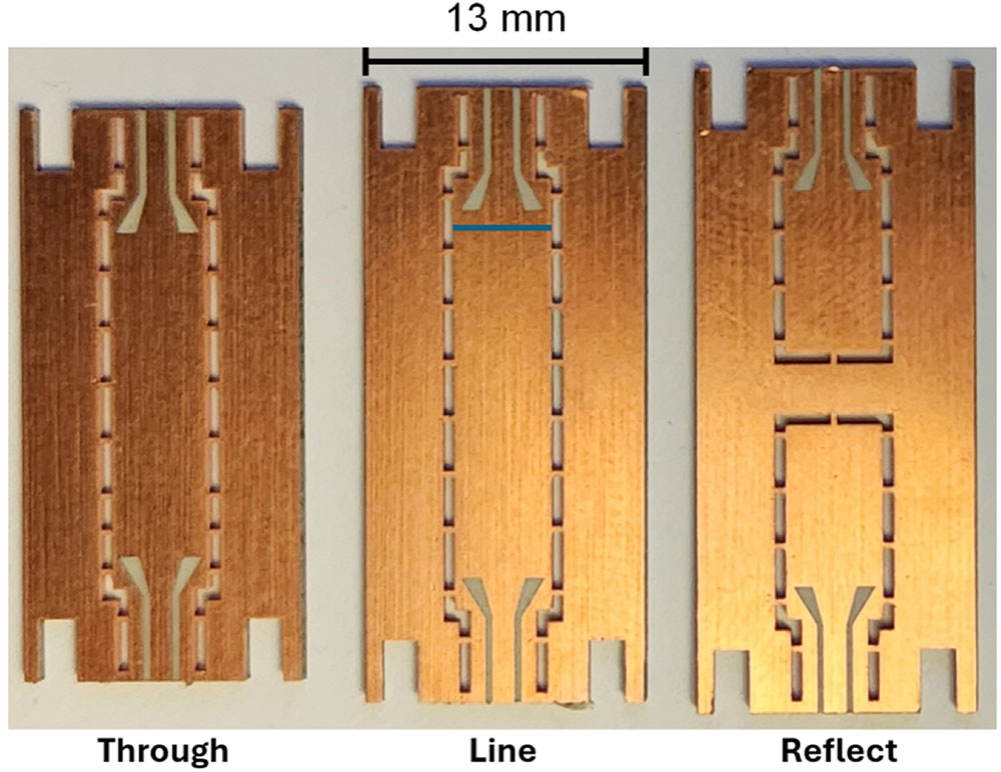
Fabricated mmWave SIW TRL calibration kit to eliminate effect of CPW lines and taper transitions, connectors, and coaxial cables in measurements conducted in this paper.

The S-parameters were measured using a 4-port Keysight vector network analyzer (VNA). Further, we have fabricated and used a set of Through, Reflect, Line (TRL) calibration kit to eliminate the effect of coaxial connection cables, connectors and the CPW to SIW transitions. The picture of the TRL calibration Kit is shown in Fig. 9.

## Data Availability

The data that support the findings of this study are available from the corresponding author upon reasonable request.

## References

[1] Jornet, J. M., Knightly, E. W. & Mittleman, D. M. Wireless communications sensing and security above 100 GHz. *Nat. Commun.* **14**, 841 (2023). 36792611 10.1038/s41467-023-36621-x PMC9931692

[2] Neuder, R. et al. Architecture for sub-100 ms liquid crystal reconfigurable intelligent surface based on defected delay lines. *Commun. Eng.* **3**, 70 (2024).

[3] Shah, S. T. et al. Coded environments: data-driven indoor localisation with reconfigurable intelligent surfaces. *Commun. Eng.* **3**, 66 (2024).

[4] Hu, K. et al. Additively manufactured flexible on-package phased array antennas for 5 G/mmWave wearable and conformal digital twin and massive MIMO applications. *Sci. Rep.* **13**, 12515 (2023). 37532806 10.1038/s41598-023-39476-w PMC10397293

[5] He, X., Cui, Y. & Tentzeris, M. M. Tile-based massively scalable MIMO and phased arrays for 5 G/B5G-enabled smart skins and reconfigurable intelligent surfaces. *Sci. Rep.* **12**, 2741 (2022). 35177671 10.1038/s41598-022-06096-9 PMC8854703

[6] Shen, G., Ma, H., Wang, X., Xu, F. & Zhu, H. “Wideband Millimeter-Wave SPST Switch in 100-nm GaN-on-Si Using Strong Mutual Coupling,” in *IEEE Transactions on Circuits and Systems II: Express Briefs*, 70, 1891-1895, (2023).

[7] Park, J., Lee, S. & Hong, S. “A 24–40 GHz Differential SPDT Switch with an NMOS and PMOS Alternating Structure and Leakage-Canceling Capacitors,”. *IEEE Trans. Circuits Syst. II: Express Briefs* **70**, 86–90 (2023).

[8] Chaillou, J. et al. “Combined role of substrate and doping on the semiconductor-to-metal transition of VO2,”. *Thin Films, ACS Appl. Electron. Mater.* **4**, 1841 (2022).

[9] Liu, C. et al. VO2 memristor-based frequency converter with in-situ synthesize and mix for wireless internet-of-things. *Nat. Commun.* **15**, 1523 (2024). 38374302 10.1038/s41467-024-45923-7 PMC10876666

[10] Singh, T., Hummel, G., Vaseem, M. & Shamim, A. “Recent Advancements in Reconfigurable mmWave Devices Based on Phase-Change and Metal Insulator Transition Materials. *” IEEE J. Microw.* **3**, 827–851 (2023).

[11] Lust, M. S., West, D. L., Smet, V., Williamson, T. G. & Ghalichechian, N. “Vanadium-Dioxide-Based Reconfigurable Ka-Band Dual-Sense Linear-to-Circular Polarizer,” in. *IEEE Trans. Antennas Propag.* **72**, 2468–2480 (2024).

[12] Qaderi, F. et al. Millimeter-wave to near-terahertz sensors based on reversible insulator-to-metal transition in VO2. *Commun. Mater.* **4**, 34 (2023). 38665394 10.1038/s43246-023-00350-x PMC11041681

[13] Bleu, Y., Bourquard, F., Barnier, V., Loir, A. S. & Garrelie, F. C. Donnet. Towards room temperature phase transition of W-doped VO2 thin films deposited by pulsed laser deposition: thermochromic, surface, and structural analysis. *Materials* **16**, 14 (2023). 10.3390/ma16010461 PMC9822253 36614799

[14] Qin, X. et al. Negative capacitors and inductors enabling wideband waveguide metatronics. *Nat. Commun.* **14**, 7041 (2023). 37923715 10.1038/s41467-023-42808-z PMC10624880

[15] Wu, G. B. et al. A universal metasurface antenna to manipulate all fundamental characteristics of electromagnetic waves. *Nat. Commun.* **14**, 5155 (2023). 37620303 10.1038/s41467-023-40717-9 PMC10449906

[16] Wu, G. B. et al. Sideband-free space–time-coding metasurface antennas. *Nat. Electron.* **5**, 808–819 (2022).

[17] Xu, G. et al. Arbitrary aperture synthesis with nonlocal leaky wave metasurface antennas. *Nat. Commun.* **14**, 4380 (2023). 37474511 10.1038/s41467-023-39818-2 PMC10359259

[18] Ivanov, A. V. et al. “Fabrication of epitaxial W-doped VO2 nanostructured films for terahertz modulation using the solvothermal process.”. *ACS Appl. Nano Mater.* **4**, 10592–10600 (2021).

[19] Xiang, W. et al. “High-quality VO2 films synthesized on polymer substrates using room-temperature pulsed laser deposition and annealing. *Ceram. Int.* **50**, 838–846 (2024).

[20] Piccirillo, C. et al. “Synthesis and characterisation of W-doped VO2 by aerosol assisted chemical vapour deposition. *Thin Solid Films* **516**, 1992–1997 (2008).

[21] Koch, D. & Mohamed, C. “The origin of the thermochromic property changes in doped vanadium dioxide.”. *ACS Appl. Mater. Interfaces* **14**, 23928–23943 (2022). 35536155 10.1021/acsami.2c02070

[22] Cassivi, Y. et al. “Dispersion characteristics of substrate integrated rectangular waveguide,”. *IEEE Microw. Wirel. Compon. Lett.* **12**, 333–335 (2002).

[23] Numan, A. B., Frigon, J.-F. & Laurin, J.-J. “Single-Pole Single-Throw Switch for Substrate-Integrated Waveguide. *” IEEE Microw. Wirel. Compon. Lett.* **28**, 221–223 (2018).

[24] Quinstar Technology Inc. Waveguide PIN Switches—Double Throw QSS/QSD Series. Accessed: Feb. 5, 2025. https://quinstar.com/shop/control-products-ferrite/pin/switches/waveguide-pin-switches-double-throw-qss-qsd/.

[25] Shepeleva, E. et al. “Low-loss K-band Photoconductive Switches in SIW Technology,” 2020 50th European Microwave Conference (EuMC), Utrecht, Netherlands, 538-541 (2021).

[26] Shepeleva, E. et al. “Integrated W-Band Photoconductive Switches in SIW Technology,” in. *IEEE Microw. Wirel. Compon. Lett.* **31**, 865–868 (2021). pp.

[27] Jones, T. R., Fisher, A., Barlage, D. W. & Peroulis, D. “A Photogenerated Silicon Plasma Waveguide Switch and Variable Attenuator for Millimeter-Wave Applications. *” IEEE Trans. Microw. Theory Tech.* **69**, 5393–5403 (2021).

[28] Wei, S.-C., Yang, C.-H., Chen, Y.-C., Chen, T.-A. & Chang, C.-Y. “V - and W -Band Substrate Integrated Waveguide (SIW) Mechanical Switch,” in. *IEEE Trans. Microw. Theory Tech.* **66**, 3090–3098 (2018).

[29] Vahabisani, N. & Daneshmand, M. “Monolithic millimeter-wave MEMS waveguide switch,”. *IEEE Trans. Microw. Theory Techn.* **63**, 340–351 (2015).

[30] Baghchehsaraei, Z. & Oberhammer, J. “Parameter analysis of millimeter-wave waveguide switch based on a MEMS-reconfigurable surface,”. *IEEE Trans. Microw. Theory Techn.* **61**, 4396–4406 (2013).

[31] Khan, S., Vahabisani, N. & Daneshmand, M. “A Fully 3-D Printed Waveguide and Its Application as Microfluidically Controlled Waveguide Switch. *” IEEE Trans. Compon., Packaging Manuf. Technol.* **7**, 70–80 (2027). pp.

[32] Chen, C.-H. & Peroulis, D. “RF Design, Power Handling, and Hot Switching of Waveguide Water-Based Absorptive Switches. *” IEEE Trans. Microw. Theory Tech.* **57**, 2038–2046 (2009).

[33] Wu, Y.-W., Qian, L., Churm, J. & Wang, Y. “Liquid metal-enabled filtering switches and switchplexers,. *IEEE Trans. Microw. Theory Techn.* **72**, 4854–4865 (2024).

